# Morphological and Physiological Traits Related to the Response and Adaption of *Bolboschoenus planiculmis* Seedlings Grown Under Salt-Alkaline Stress Conditions

**DOI:** 10.3389/fpls.2021.567782

**Published:** 2021-03-05

**Authors:** Yu An, Yang Gao, Shouzheng Tong, Bo Liu

**Affiliations:** ^1^Northeast Institute of Geography and Agroecology, Chinese Academy of Sciences, Changchun, China; ^2^Jilin Academy of Agricultural Science, Changchun, China

**Keywords:** *Bolboschoenus planiculmis*, early establishment, salt-alkaline stress, growth performance, wetland plant

## Abstract

Soil saline-alkalization is expanding and becoming a serious threat to the initial establishment of plants in inland salt marshes on the Songnen Plain in Northeast China. *Bolboschoenus planiculmis* is a key wetland plant in this area, and its root tubers provide food for an endangered migratory Siberian crane (*Grus leucogeranus*). However, the survival of this plant in many wetlands is threatened by increased soil saline-alkalization. The early establishment of *B. planiculmis* populations under salt and alkaline stress conditions has not been well understood. The aim of this study was to investigate the response and adaption of the seedling emergence and growth of *B. planiculmis* to salt-alkaline mixed stress. In this study, *B. planiculmis* root tubers were planted into saline-sodic soils with five pH levels (7.31–7.49, 8.48–8.59, 9.10–9.28, 10.07–10.19, and 10.66–10.73) and five salinity levels (40, 80, 120, 160, and 200 mmol⋅L^–1^). The emergence and growth metrics, as well as the underlying morphological and physiological traits in response to salt-alkaline stress were explored for 2-week-old seedlings. The seedling emergence, growth, and leaf and root traits showed distinct responses to the pH and salt gradients. Under the lower saline-alkaline condition (pH ≤ 9.10–9.28 and salinity ≤ 80 mmol⋅L^–1^), the seedling growth was substantially facilitated or not significantly altered. Salinity affected the seedlings more significantly than alkalinity did. In particular, among the salt ions, the Na^+^ concentration had predominantly negative effects on all the morphological and physiological traits of the seedlings. Seedling emergence was more tolerant to salinity and, based on its observed close relationships with pH and the alkaline ion CO_3_^2–^, was highly alkalinity-dependent. Moreover, the leaf area and photosynthetic rate, as well as the root surface area and tip number mainly accounted for the response of the seedling biomass to salt-alkaline stress. This is evidence of the adaption of *B. planiculmis* to saline-alkaline conditions largely due to the responses of its morphological and physiological traits. This study provides a mechanistic process-based understanding of the early seedling establishment of *B. planiculmis* populations in response to increased soil saline-alkalization in natural wetlands.

## Introduction

Soil saline-alkalization is a major cause of land degradation worldwide (Ma et al., 2015). Parent materials, climate, topography, and anthropogenic activities contribute to the formation and evolution of soil saline-alkalization (Liu et al., 2009). There are large areas of saline-alkaline land in China and the Songnen Plain has one of the largest areas of saline-alkaline land in Northeast China (approximately 4 × 10^6^ ha) (Wang et al., 2009). The ecological functions of this area have become severely degraded because of increased soil saline-alkalization. As a result, it has become an ecologically fragile area, and is an important area for maintaining national ecological security (Yang et al., 2010). Among the salts in soil, NaCl and Na_2_SO_4_ are the main neutral components and Na_2_CO_3_ and NaHCO_3_ are the main causes of saline-alkaline conditions (Yang et al., 2011). Compared with neutral salts, NaHCO_3_ and Na_2_CO_3_ are more harmful to vegetation because of the combined effects of high electrical conductivity and high pH (Tang and Turner, 1999; Shi and Sheng, 2005; Chen et al., 2017). Consequently, salinity and alkalinity act as the key attributes affecting plant establishment, growth, and distribution in saline-alkaline soils (Silvestri et al., 2005; An et al., 2019).

During the plant life cycle, the initial phase (e.g., seedling emergence) can be widely used to assess the adaptive ability of the plant to increased alkalinity in the soil (Stokes et al., 2011). However, there are differences in the pH thresholds among plant species. Some can successfully emerge over a wide pH range, while others only emerge at a certain pH (Nakamura and Hossain, 2009; Ebrahimi and Eslami, 2012). During plant regeneration, vegetative propagation is the main mode of reproduction of aquatic plants in natural conditions (Sosnová et al., 2010). Aquatic macrophytes commonly and easily propagate through stems, leaves, or roots, and can colonize new areas quickly and efficiently (Mony et al., 2011). The early stage of the establishment of these species, which includes seedling emergence and growth, is vulnerable to environmental changes (Engels et al., 2011). Variations in the seedling emergence of plants under stress conditions may reveal their adaptive strategies and could be used to evaluate the effects of stress factors (Lowe et al., 2010). To date, studies of wetland plants have only focused on the effect of saline and alkaline stress on seed germination, seedling and vegetative growth (Liu et al., 2018; Wang et al., 2020), evaluations of the vegetative propagation of typical wetland plants under mixed stress conditions are still rarely reported.

Plants growing in high-salt soil usually face problems related to osmotic and ionic stresses (Lv et al., 2013). High alkalinity affects the availability of some mineral nutrients in soil, resulting in nutrient deficiency for plants (Shi and Wang, 2005; Li et al., 2010). Additionally, high pH may suppress ion absorption in plant cells, consequently destroying their ion homeostasis (Yang et al., 2009). The influences of salt concentration and composition, pH, and their combinations on the growth and development of plants have been investigated and compared (Shi and Wang, 2005; Yang et al., 2011; Zhang et al., 2018). Likewise, plants have several response strategies for averting the negative effects of salt-alkaline stress (Liu et al., 2015). The ecophysiological mechanisms underlying plants’ responses and adaptations to salt or alkaline conditions have been widely reported (Elmore et al., 2006). For instance, the photosynthetic characteristics of plant seedlings under increased alkalinity can provide important information for understanding the adaptive response of plants, which directly determine their productivity and acclimation in natural environments (Yang et al., 2011). In addition, the morphological characteristics of plants, such as leaf traits, can also be used as indicators in plants subjected to salt and alkaline conditions (Bernstein et al., 2010; Rouphael et al., 2012). Furthermore, plant roots are the major organ in direct contact with the soil and thus primarily encounter any abiotic stresses (Yang et al., 2011). The inherent variations in root architecture allow plants to adapt to saline-alkaline conditions, ultimately resulting in greater efficiency in nutrient and water absorption (An et al., 2019). Plants attempt to circumvent highly saline media by altering the direction of their root growth (Galvan-Ampudia et al., 2013). However, studies to date have mainly focused on the response and adaption of crop and forage plants to salt-alkaline stress; the morphological and physiological strategies of typical wetland plants to mixed stress remain poorly understood.

Plants of genus *Bolboschoenus* are commonly distributed in saline wetlands, and often occupy a wide range of habitats, including both coastal and inland salt marshes (Ljevnaić-Mašić et al., 2020). The ecological properties of these species such as their dispersal abilities and distribution as well as their relationships with habitat conditions, have been are investigated (Hroudová et al., 2007; Píšová et al., 2017). *Bolboschoenus maritimus*, for example, is regarded as a halophyte usually inhabiting saline habitats. A prominent response of this species to salinity is a change in biomass allocation from shoots to tubers (Morris and Ganf, 2001). Its adaptive ability to salinity is largely due to stomata that is less sensitive to environmental salinity (Maricle and Maricle, 2018). Meanwhile, comparisons of the salinity tolerance of *B. planiculmis* to that of other *Bolboschoenus* species indicates that the broader extent of its phenotypic plasticity enables it to inhabit and extend its range to saline habitats (Hroudová et al., 2014). It has also been reported that both *B. yagara* and *B. planiculmis* communities are not adaptive to saline habitats, but are able to become established in eutrophic freshwater habitats and those with a fluctuating water level, as well as in arable land with a rich nutrient supply (Ljevnaić-Mašić et al., 2020). Thus, the ecological adaptation mechanism that occurs in *Bolboschoenus* species growing in different conditions is not precisely known.

*Bolboschoenus planiculmis* is often found in the saline-sodic wetlands on the western Songnen Plain, where it mainly reproduces and expands its population using vegetative propagules (An et al., 2018). The *B. planiculmis* wetlands on the Songnen Plain can provide a habitat for the migratory Siberian crane (*Grus leucogeranus*), and the root tubers of this plant are a food source for this waterfowl. However, these wetlands are suffering from increased soil salinization and alkalinization, and the vegetation is being degraded (An et al., 2018). Existing studies on the growth of *B. planiculmis* in this area under salt stress have, to our knowledge, only investigated either NaCl or water management (Zhang et al., 2014; An et al., 2018). Studies of the effects of saline-sodic stress on *B. planiculmis* are still lacking, greatly hindering our understanding of this species’ ecological limits. The aims of this study were to (1) investigate the responses of *B. planiculmis* seedling variations to mixed salt-alkaline stresses, (2) evaluate the dominant stress factors controlling *B. planiculmis* seedlings, and (3) clarify the underlying morphological and physiological traits potentially responsible for the response and adaptive strategies of *B. planiculmis* seedlings to stress. This study can provide valuable implications for the survival and early establishment of *B. planiculmis* populations in saline-alkali environments, as well as for designing effective restoration projects.

## Materials and Methods

### Plant and Soil Materials

Plant and soil materials were collected from the Ertou Wetland (45°53′N; 123°38′E) located in the buffer zone of Momoge National Nature Reserve on the western Songnen Plain. In late April 2018, the roots of *B. planiculmis* were excavated within a soil depth of 30 cm and washed with tap water. After removing the rhizomes and fibrous roots, the root tubers were then stored at 4°C in damp and dark conditions. Surface soil (30 cm depth) from the flood plain of the Nengjiang River was collected and used as a potting medium in the experiment. The soil is a meadow bog soil with a pH of 7.11, electrical conductivity of 277 μS cm^–1^, organic matter content of 22.4 g kg^–1^, total nitrogen content of 2.55 g kg^–1^, and total phosphorus content of 0.36 g kg^–1^. Before use, the soil was air-dried, crushed and sieved through a 2 mm sieve to remove gravel and organic particles.

### Design of Simulated Saline-Alkaline Conditions

A completely randomized experimental design was used to study the effects of soil salinity and pH. According to the salt components in the salt-alkaline soils investigated by a previous study (Ge and Li, 1990), NaCl, Na_2_SO_4_, Na_2_CO_3_, and NaHCO_3_ were selected and mixed in various molar proportions to reflect the salinity and alkalinity ranges in natural soil. Five treatment groups were set with increasing pH (ranging from 7.31 to 10.73). The salt compositions of the five treatment groups (labeled as A, B, C, D, and E) are presented in Table 1. There were five levels of salinity (40, 80, 120, 160, and 200 mmol L^–1^), in each group, for a total of 25 salt-alkaline stress treatments with varying salinity and pH. Details of the treatments (labeled as A1, A2, A3…E3, E4, E5) are illustrated in Table 2. Stress factors such as ions concentrations were calculated on the basis of the salt molar ratios listed in Table 1 and the given salinity. The treatment without salt addition was considered to be the control (CK). All salt-alkaline stress treatments and CK were replicated three times.

**TABLE 1:** Salt compositions and their molar ratios in the treatment groups.

Treatment groups	NaCl	Na_2_SO_4_	NaHCO_3_	Na_2_CO_3_
A	1	1	0	0
B	1	2	1	0
C	2	8	9	1
D	1	1	1	1
E	8	2	2	8

*A, pH 7.31–7.49; B, pH 8.48–8.59; C, pH 9.10–9.28; D, pH 10.07–10.19; E, pH 10.66–10.73.*

**TABLE 2:** The stress factors evaluated in the various treatments.

	pH	Salinity (mmol L^–1^)	[Na^+^] (mmol L^–1^)	[Cl^–^] (mmol L^–1^)	[SO_4_^2–^] (mmol L^–1^)	[HCO_3_^–^] (mmol L^–1^)	[CO_3_^2–^] (mmol L^–1^)
A1	7.31	40	30	20	20	0	0
B1	8.48	40	60	10	20	10	0
C1	9.10	40	58	4	16	18	2
D1	10.14	40	60	10	10	10	10
E1	10.66	40	60	16	4	4	16
A2	7.49	80	60	40	40	0	0
B2	8.58	80	120	20	40	20	0
C2	9.20	80	116	8	32	36	4
D2	10.19	80	120	20	20	20	20
E2	10.73	80	120	32	8	8	32
A3	7.48	120	90	60	60	0	0
B3	8.58	120	180	30	60	30	0
C3	9.28	120	174	12	48	54	6
D3	10.19	120	180	30	30	30	30
E3	10.73	120	180	48	12	12	48
A4	7.34	160	120	80	80	0	0
B4	8.59	160	240	40	80	40	0
C4	9.22	160	232	16	64	72	8
D4	10.15	160	240	40	40	40	40
E4	10.71	160	240	64	16	16	64
A5	7.46	200	150	100	100	0	0
B5	8.55	200	300	50	100	50	0
C5	9.16	200	290	20	80	90	10
D5	10.07	200	300	50	50	50	50
E5	10.69	200	300	80	20	20	80

### Growth Conditions

The experiment started on May 1, 2018. Plastic pots (15 cm diameter × 10 cm depth) were used in this study and each pot was filled with the potting medium (500 g pot^–1^). There were 78 pots in total (25 salt-alkaline treatments and CK with three repetitions per treatment). Thirty fully developed tubers of *B. planiculmis* of uniform size (mean dry weight 1.2 g) were selected and planted into the soil (5 cm). The pots were watered with a saline-alkaline solution to 50% soil water content to avoid inhibition of root tuber emergence and seedling growth of *B. planiculmis* due to flooding (An et al., 2018). All the pots were then placed into the growth chambers with a 12-h photoperiod (500 μmol m^–2^ s^–1^ of photosynthetically active radiation), 25/20°C day/night temperature, and 70–75% relative humidity. All the pots were rotated randomly to standardize daily light exposure and the evaporated water in the pots was resupplied daily with distilled water after weighing.

### Aerial Part Measurements

Seedling emergence was recorded daily when sprouts were 1 cm above the soil surface. The emergence rate was calculated by dividing the number of emerged seedlings by the number of tubers planted in each pot and multiplying the product by 100. On May 15 (2 weeks after planting), the seedling height was measured as the distance from the soil surface to the top of stem. Three exposed leaves in each pot were selected for the leaf area measurements using a leaf area meter (Li-Cor 3100, Li-Cor). All the selected leaves were then collected and dried at 60°C for 72 h to a constant weight. The mean of the leaf traits was calculated for each pot. Aerial parts of the plants in each pot were removed at the soil surface level and dried at 90°C for 1 h and then at 60°C to determine the shoot biomass. The shoot biomass was calculated as the sum of the aerial plant parts (leaves + stems). Individual plant shoot biomass was computed as the ratio of total shoot dry weight/the number of emerged seedlings in each pot.

### Photosynthetic Trait Measurements

Before sampling, the expanded young leaves were selected to measure the light-saturated net photosynthetic rate (*Pn*), transpiration rate (*Tr*), and stomatal conductance (*Gs*) using a portable gas-exchange system (Li-6400, Li-Cor, Lincoln, NE, United States) with an integrated light source (6200-02BLED, Li-Cor). The photosynthetic photon flux density in the leaf chamber was 1500 μmol m^–2^ s^–1^. The vapor pressure deficit was kept at 1.0–1.5 kPa. The air temperature was set at 25°C, and the ambient CO_2_ concentration was set at 360 μmol mol^–1^. The instantaneous water use efficiency (WUE) of the leaves was calculated as WUE = *Pn*/*Tr*. Three to five leaves were measured per pot, and the mean was calculated for each pot.

### Root Trait Measurements

Roots were removed from each pot and rinsed using tap water. The fine roots remaining in the soil were isolated by overfilling the soil with water and passing the outflow through a 0.25 mm mesh sieve to collect the roots. All the roots in each pot were oven-dried at 60°C to a constant weight. Individual plant root biomass was computed as the ratio of the total root dry weight/the number of emerged seedlings in each pot. Before drying, one to three intact roots in each pot were randomly selected and scanned at 400 dpi resolution. Finally, the root length, root mean diameter, root surface area, and root tip number were determined using the WinRHIZOPRO (version 2003b) root analyzing system (Regent Instruments Inc., Quebec, Canada). The means of the root traits from one pot indicated the data for a replication of the treatment.

### Data Analysis

The effects of pH and salinity on emergence, growth, and morphological and physiological traits of *B. planiculmis* seedlings were determined by two-way ANOVA at *p* ≤ 0.05, *p* ≤ 0.01, and *p* ≤ 0.001 levels. The above analyses were performed with SPSS software (ver.18.0, Chicago, IL, United States). Principle component analysis (PCA) was performed on the growth as well as the morphological and physiological measurements of the treatments using R Studio (ver. 1.2.1335), and the variance proportion was analyzed using JMP Genomics (ver. 14). A redundancy analysis (RDA) was conducted to quantify and test the effects of the salt factors on *B. planiculmis* seedling variations. The variables were centered and standardized by applying a square root transformation and then a logarithmic transformation. The significance levels of the explanatory variables were determined using Monte Carlo tests (499 permutations). The RDA was performed using the Canoco 4.5 software package (Microcomputer Power, Ithaca, NY, United States). Pearson’s correlation analysis was performed to examine the relationships between the stress variables and the emergence and growth performance of the *B. planiculmis* seedlings. The correlation coefficients (*r*) were considered significant at the *p* ≤ 0.05, *p* ≤ 0.01, and *p* ≤ 0.001 levels. Stepwise multiple linear regressions were employed to determine the morphological and physiological traits that best predicted seedling shoot and root biomasses. The final multiple regression model contained only the significant variables (*p* ≤ 0.05). The regression coefficients (*R*^2^) were considered significant at the *p* ≤ 0.05, *p* ≤ 0.01, and *p* ≤ 0.001 levels. All the correlations and regressions were analyzed using SPSS Version 18.0.

## Results

### Effects of Salt-Alkaline Stresses on *B. planiculmis* Seedlings

**FIGURE 1:**
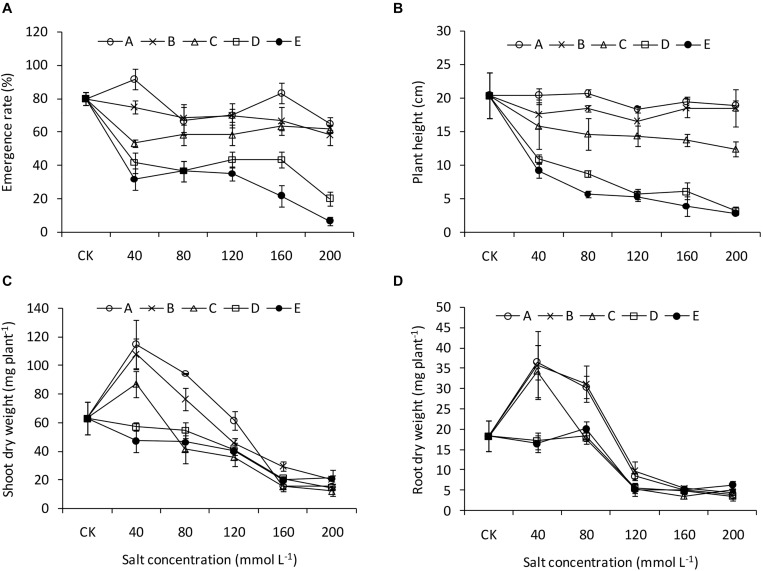
Emergence and growth performance of *B. planiculmis* seedlings under salt-alkaline mixed stresses. **(A)** Emergence rate. **(B)** Plant height. **(C)** Shoot biomass. **(D)** Root biomass. Vertical lines are error bars.

**FIGURE 2:**
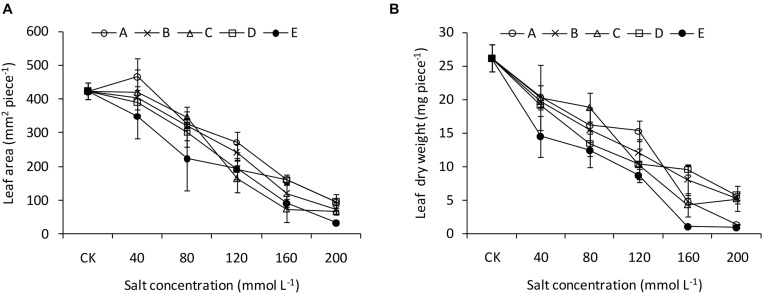
Leaf traits of *B. planiculmis* seedlings under salt-alkaline mixed stresses. **(A)** Leaf area. **(B)** Leaf dry weight. Vertical lines are error bars.

The two-way ANOVA revealed significant effects of pH and salinity and their interactions on almost all the seedling traits of *B. planiculmis*, with a few exceptions (Table 3). The effects of pH and salinity interactions on the leaf area, transpiration rate, root length, and root average diameter were not significant. The results indicated that seedling emergence was sensitive to salt-alkaline stress (Figure 1A). Compared with CK, treatment A1 had a positive effect on the seedling emergence, and the emergence rate decreased as expected with increased salinity and alkalinity. Moreover, the emergence rate dropped remarkably as the salinity increased at a higher pH. However, the emergence rate dropped less than 50% in the D and E groups regardless of salinity level. The emergence rates of the D5 and E5 treatments were 20.0 and 6.7%, respectively. Similarly, plant height remained relatively stable in the A, B, and C groups over the salinity gradient and decreased as both salinity and pH increased (Figure 1B). Under the lower condition (pH less than 8.48–8.58 and salinity less than 80 mmol L^–1^), the shoot and root biomasses were greater than CK and decreased as salinity and pH increased (Figures 1C,D). The effects of salt-alkali stress on leaf area and leaf dry weight were significant (Figure 2). Although the A1 treatment increased the leaf area compared with CK (Figure 2A), the leaf area decreased sharply as the salinity and alkalinity increased. Leaf dry weight also significantly decreased with salt-alkali stress (Figure 2B), and decreased with increased salt concentration.

**TABLE 3:** Results (*F*-values) of the two-way variance analysis (ANOVA) of pH and salinity for the emergence and growth performance of the *B. planiculmis* seedlings.

Dependent variable	pH	Salinity	pH *×* Salinity
Emergence rate	133.2***	12.3***	3.7***
Plant height	223***	11.2***	2.2*
Shoot dry weight	34.3***	194.7***	10.5***
Leaf area	6.7***	125.4***	1.2^ns^
Leaf dry weight	8.8***	95.7***	2.0*
Photosynthetic rate	24.0***	207.5***	3.1***
Stomatal conductance	10.8***	107.7***	2.2*
Transpiration rate	11.2***	94.3***	0.9^ns^
Water use efficiency	16.2***	44.9***	5.6***
Root dry weight	15.9***	171.4***	6.5***
Root length	7.6***	64.9***	1.3^ns^
Root surface area	7.5***	92.5***	2.0*
Root average diameter	1.5^ns^	18.1***	1.4^ns^
Root tips number	18.4***	281.3***	10.5***
df	4	4	16

ns, not significant. *p < 0.05, **p < 0.01, and ***p < 0.001.

**FIGURE 3:**
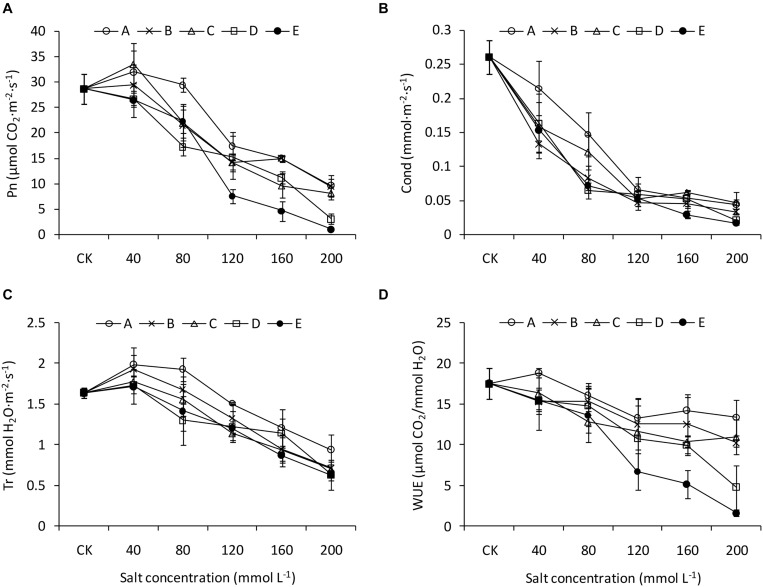
Photosynthetic traits of *B. planiculmis* seedlings under salt-alkaline mixed stresses. **(A)** Photosynthetic rate (*Pn*). **(B)** Transpiration rate (*Tr*). **(C)** Stomatal conductance (*Gs*). **(D)** Water use efficiency (WUE). Vertical lines are error bars.

The *Pn* of the leaves decreased with increasing salinity and alkalinity, although it was greater than that of CK when at low salinity and alkalinity (Figure 3A). Compared with that of CK, *Gs* decreased sharply under the saline-alkaline conditions, especially at greater alkalinity. However, at the greater salinity levels (120 mmol L^–1^, 160 mmol L^–1^, and 200 mmol L^–1^), the *Gs* values were similar among the different treatments (Figure 3B). *Tr* also decreased with increasing salinity and alkalinity without interactions between salinity and alkalinity (Figure 3C). Salinity and alkalinity had negative influences on WUE, and these influences were more pronounced when high salinity and high alkalinity were combined (Figure 3D).

**FIGURE 4:**
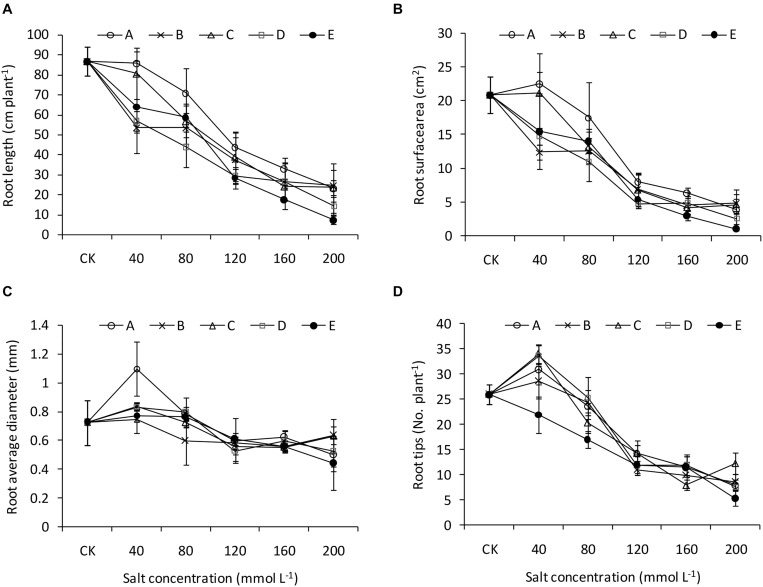
Root traits of *B. planiculmis* seedlings under salt-alkaline mixed stresses. **(A)** Root length. **(B)** Root surface area. **(C)** Root average diameter. **(D)** Root tip number. Vertical lines are error bars.

The root length and surface area generally decreased with increasing salinity and alkalinity (Figures 4A,B). Under low salinity and alkalinity treatments, the root average diameter and tips number were similar to or greater than those of CK. Although the increment exited in the A1 treatment, the root diameter was similar among treatments with high salinity and alkalinity (Figure 4C). The A1, B1, C1, and D1 treatments significantly increased the root tip number compared with CK (Figure 4D).

### Evaluation of Stress Factors on *B. planiculmis* Seedlings

**FIGURE 5:**
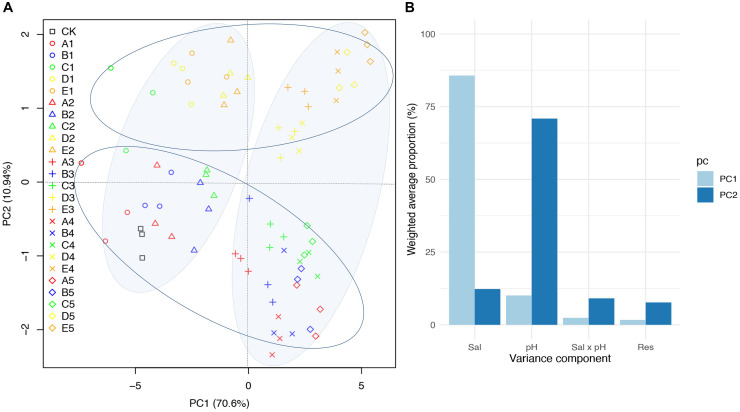
Principal component analysis (PCA) of seedling performance of *B. planiculmis* treated with different salinity and pH combinations. **(A)** Score scatter plot of PC1 versus PC2. **(B)** Variance decomposition of PC1 and PC2 as implemented in JMP Genomics. CK, control; Sal, salinity. The details for the treatments in **(A)** are shown in Table 2.

The PCA of the measurements of the *B. planiculmis* seedlings showed that the first component (PC1: 70.6%) and second component (PC2: 10.94%) explained 81.54% of the total variance (Figure 5A). The score plot of the PCA clearly divided the CK and low-salinity treatments (treatments 1 and 2) from the high-salinity treatments (treatment 3, 4, and 5) along PC1, and also divided the CK and low-pH treatments (treatments A, B, and C) from the high-pH treatments (treatments D and E) along PC2. The variation attributable to salinity accounted for 85.7% of the variation in PC1 while the variation attributable to pH accounted for 70.9% of the variation in PC2 (Figure 5B).

The linear regression indicated that the emergence rate and plant height were negatively correlated with pH (*p* < 0.001), Na^+^, SO_4_^2–^, and CO_3_^2–^ (*p* < 0.05) (Table 4). There were significant correlations between Na^+^ and all seedling traits. The relationships between the other stress factors and shoot biomass and leaf area were significant (*p* < 0.05). The leaf dry weight was closely related to salinity, Na^+^, Cl^–^, SO_4_^2–^, and CO_3_^2–^ (*p* < 0.05). *Pn*, *Gs*, and *Tr* were significantly correlated with salinity and salt ions (*p* < 0.05). WUE was significantly correlated with salinity, Na^+^, Cl^–^, and CO_3_^2–^ (*p* < 0.05). Root biomass and root tip number were negatively correlated with five factors (salinity, Na^+^, Cl^–^, SO_4_^2–^, and CO_3_^2–^) (*p* < 0.05). In addition, the root length and root surface area were significantly correlated with salinity and salt ions. The relationships between root diameter and salinity, Na^+^, Cl^–^, and SO_4_^2–^ were significant (*p* < 0.05).

**TABLE 4:** Correlation coefficients (*r*) between stress factors and emergence, growth, and morphological and physiological traits of *B. planiculmis* seedlings.

	pH	Salinity	[Na^+^]	[Cl^–^]	[SO_4_^2–^]	[HCO^3–^]	[CO_3_^2–^]
Emergence rate	−0.893***	−0.211^ns^	−0.420*	−0.158^ ns^	0.466*	−0.068^ns^	−0.872***
Plant height	−0.927***	−0.204^ns^	−0.433*	−0.079^ ns^	0.508**	−0.155^ns^	−0.905***
Shoot dry weight	−0.353^ns^	−0.834***	−0.794***	−0.487*	−0.483*	−0.519**	−0.401*
Leaf area	−0.221^ns^	−0.951***	−0.904***	−0.602***	−0.563**	−0.535**	−0.449*
Leaf dry weight	−0.206^ns^	−0.915***	−0.815***	−0.712***	−0.505***	−0.362^ns^	−0.490*
Photosynthetic rate	−0.332^ns^	−0.907***	−0.899***	−0.598**	−0.414*	−0.475*	−0.593**
Stomatal conductance	−0.255^ns^	−0.857***	−0.836***	−0.574**	−0.485*	−0.433*	−0.450*
Transpiration rate	−0.299^ns^	−0.937***	−0.944***	−0.524**	−0.519**	−0.602***	−0.486*
Water use efficiency	−0.370^ns^	−0.719***	−0.723***	−0.612***	−0.108^ns^	−0.213^ns^	−0.768***
Root dry weight	−0.275^ns^	−0.822***	−0.759***	−0.527**	−0.498*	−0.453*	−0.376^ns^
Root length	−0.296^ns^	−0.899***	−0.886***	−0.585**	−0.465*	−0.475*	−0.523**
Root surface area	−0.242^ns^	−0.888***	−0.856***	−0.578**	−0.503**	−0.471*	−0.458*
Root average diameter	−0.199^ns^	−0.778***	−0.729***	−0.541**	−0.457*	−0.376^ns^	−0.384^ns^
Root tips number	−0.119^ns^	−0.871***	−0.789***	−0.634***	−0.549**	−0.387^ns^	−0.383^ns^

ns, not significant. *p < 0.05, **p < 0.01, and ***p < 0.001.

**FIGURE 6:**
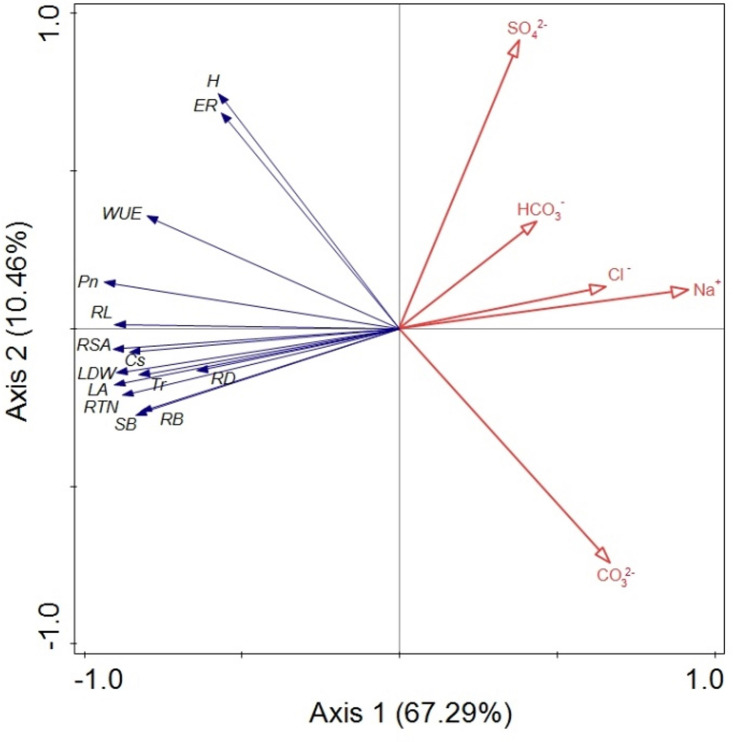
Bioplot diagram of the redundancy analysis (RDA) on *B. planiculmis* constrained by Na^+^, Cl^–^, SO_4_^2–^, HCO_3_^–^, and CO_3_^2–^. ER, emergence rate; H, plant height; LA, leaf area; LDW, leaf dry weight; SB, shoot biomass; *Pn*, photosynthetic rate; *Tr*, transpiration rate; *Gs*, stomatal conductance; WUE, water use efficiency; RB, root biomass; RL, root length; RTN, root tip number; RSA, root surface area; RD, root average diameter. The total explained variance by all the selected soil variables is 79.7% (*F* = 55.3, *p* = 0.002). Axis 1 and axis 2 explained 67.3 and 10.5% of the variation, respectively, in the *B. planiculmis* seedlings.

The RDA was performed to quantify the effects of five salt variables (Na^+^, Cl^–^, SO_4_^2–^, HCO_3_^–^, and CO_3_^2–^) on *B. planiculmis* seedling variations based on the data from the various treatments (Figure 6). Eigenvalues for the first, second, third, and fourth axes were 0.673, 0.105, 0.014, and 0.005, respectively. The five salt variables explained 79.7% of the *B. planiculmis* seedling variations (Monte Carlo test with 499 repetitions, *F* = 55.3, *p* = 0.002). In addition, the RDA showed a strong correlation between vegetation and environmental factors with species–environment correlations of 0.973 on the first axis and 0.941 on the second axis. In particular, Na^+^ negatively affected emergence, growth, and all the morphological and physiological traits. Emergence was negatively correlated with CO_3_^2–^ (Figure 6). The marginal effects and conditional effects on the eigenvalue of the explained variance indicated that Na^+^ was the best explanatory variable (57%, *F* = 95.2, *p* = 0.002) (Table 5). The conditional effects showed that the additional variables, CO_3_^2–^ (12%, *F* = 30.36, *p* = 0.002), SO_4_^2–^ (9%, *F* = 25.55, *p* = 0.002), HCO_3_^–^ (2%, *F* = 5.49, *p* = 0.002), and Cl^–^ (0%, *F* = 2.87, *p* = 0.012) could explain the statistically significant amount of variation.

**TABLE 5:** Marginal and conditional effects on the eigenvalue obtained from the summary of forward selection using a Monte Carlo Permutation test.

		Marginal Effects	Conditional Effects		
Variable	Var.N	Lambda1	LambdaA	*F*	*P*
Na^+^	1	0.57	0.57	95.20	0.002
CO_3_^2–^	5	0.36	0.12	30.36	0.002
Cl^–^	2	0.30	0.00	2.87	0.012
SO_4_^2–^	3	0.18	0.09	25.55	0.002
HCO_3_^–^	4	0.15	0.02	5.49	0.002

### Morphological and Physiological Traits as Predictors of Seedling Biomass Production

The correlations between the underlying morphological and physiological traits and the seedling biomass production were evaluated with multiple linear regressions (Table 6). The results showed that there were two traits significantly correlated with shoot biomass and root biomass (*R*^2^ = 0.718 and 0.741, *p* < 001, respectively). The highest proportion of variation in seedling shoot biomass was explained by the variation in photosynthetic rate and leaf area (*p* < 0.001). The root surface area and root tip number accounted for the variations in root biomass (*p* < 0.001).

**TABLE 6:** Morphological and physiological traits as predictors of seedling shoot and root biomass in response to salt-alkaline stress determined by a linear stepwise multiple regression analysis.

Morphological and physiological traits	Standardized coefficients	Shoot biomass	Morphological and physiological traits	Standardized coefficients	Root biomass
Plant height		ns	Root length		ns
Leaf area	0.438	3.6***	Root surface area	0.487	4.2***
Leaf dry weight		ns	Root average diameter		ns
Photosynthetic rate	0.446	3.8***	Root tip number	0.409	3.5**
MS regression		25120.5	MS regression		3759.7
MS residual		264.1	MS residual		35.2
*R*^2^		0.718***	*R*^2^		0.741***

F-values and significance levels are shown for traits with a significant effect. Mean squares for the final regression equations (containing the “significant variables” with standardized coefficients), and proportion of variance explained by the model (R^2^) are indicated for both biomasses. ns, not significant. **p < 0.01, ***p < 0.001.

## Discussion

The growth of some salt-tolerant plant species is moderately suppressed, or even improved under lower salinity conditions (Bose et al., 2014). A similar tendency was observed in this study. Some seedling metrics of *B. planiculmis*, including emergence, biomass, and leaf and root traits, that were actually facilitated or not significantly altered by low salinity-alkalinity (treatments with pH ≤ 9.10–9.28 and salinity ≤ 80 mmol L^–1^) in comparison with those without stress, confirming its adaptive ability to salt-alkaline stress. During adaptive evolution in natural wetlands, the *B. planiculmis* populations living in the salt-sodic environments have retained their tolerance to salt, which probably developed their varied tolerances to pH stress. The results of previous studies on *B. maritimus* correspond to our results, indicating that salt-alkaline conditions, with higher pH and conductivity are favorable for *B. maritimus* development, implying its potential for adapting saline and alkaline habitats (Hroudová et al., 2014; Jenačković et al., 2016). This indicates that it may be more appropriate to classify *B. planiculmis* as a salt-alkali plant, and that a degree of adaptability to saline -alkaline condition is crucial for the early establishment of a *B. planiculmis* population.

As might be expected, the growth of *B. planiculmis* seedlings was suppressed under soil conditions with higher levels of salt concentration. The growth inhibition of seedlings from seed germination was also reported by a previous study on other wetland plants (Wang et al., 2020). Salt stress interferes with plant growth by causing reduced water potential around the plant rhizosphere and ionic toxicity, resulting from turbulence in specific ion concentrations inside the root tissue (Ghoulam et al., 2002; de Lacerda et al., 2003; Sosa et al., 2005). As has been found in natural populations of *Scirpus planiculmis* (=*B. planiculmis*), the root biomass of *B. planiculmis* was negatively correlated with pH, and a high pH significantly affected its growth biomass allocation and rhizome morphology (Ning et al., 2014). The results of this study also demonstrated that total salinity affected the *B. planiculmis* seedlings more than alkaline stress. This can be explained by the species-specific responses of plants to the stress factors when subjected to mixed stresses (Guo C. Y. et al., 2015). However, the opposite result was observed in grassland plants, in which alkaline stress had more harmful effects on the plants than salt stress (Yang et al., 2008; Zhang et al., 2018). This is likely because drought stress occurs in combination with salt and alkaline stresses in grasslands, causing a synergistic enhancement in soil alkalinization. In salt-sodic wetlands, optimal water conditions could reduce soil salinization and alkalinization, and mitigate the intensity of salt-alkaline stress on wetland plants (An et al., 2019).

In addition to the negative effects on vegetative growth, salts in soil may create a high osmotic potential for plant propagules, preventing their water absorption (Khajeh-Hosseini et al., 2003; Sosa et al., 2005). The root tubers of *B. planiculmis* used serve as the main vegetative propagules in natural habitats (An et al., 2018). In this study, the emergence rate of the *B. planiculmis* tubers was highly correlated with pH, demonstrating that the alkaline salts inhibited seedling emergence more greatly than the neutral salts did. This may be because high pH around the rhizosphere of propagules inhibits reproduction (Zhang et al., 2015). In the salt-sodic soils, Na_2_CO_3_ and NaHCO_3_ accumulations mainly accounted for the high pH values, because both salts are soluble and can undergo alkaline hydrolysis (Chi et al., 2012). The intense inhibitory effects of CO_3_^2–^ and HCO_3_^–^ on plant seedling emergence were possibly attributed to their buffer capacity. The results of the RDA also confirmed that emergence rate was closely related to CO_3_^2–^. Thus, the pH attributed to CO_3_^2–^ and its buffer effects, not salt concentration, should be the restriction factor of seedling emergence of *B. planiculmis*. An emergence assessment of *B. planiculmis* tubers under controlled conditions can predict the response of *B. planiculmis* population density to a specific component of complex alkaline stress syndrome.

Stress tolerance varies depending on the plant species and soil conditions. In particular, salt tolerance usually depends on the ion’s absorption dose and allocation in plants (Flowers and Colmer, 2008). There are some tolerant mechanisms for halophytes under salt stress. For example, excessive accumulation of Na^+^ within the vacuoles in epidermal bladder cells enables salt stress adaptation (Zhao et al., 2020). A previous study conducted in saline-sodic soil revealed that high Na^+^ concentration not only influenced seed germination but also seedling growth of plant (Zhao et al., 2014). In this study, Na^+^ concentration played an important role in influencing the morphological and physiological traits of the *B. planiculmis* seedlings, such as growth, photosynthetic performance, and leaf and root traits. This result is consistent with several previous studies. High Na^+^ concentrations negatively affect germination and vegetative growth of plants, although low Na^+^ concentrations can adjust osmolarity in the vacuole (Wang et al., 2002; Zhang et al., 2010). Increased salt concentrations in soil lead to large amounts of Na^+^ to be taken up by roots. Na^+^ consequently impairs metabolic processes via ion toxicity and osmotic effects on water uptake or interference with the uptake of nutrients (Grewal, 2010; Deinlein et al., 2014). Plants tend to enact some mechanisms to mitigate Na^+^ stress by reducing physiological traits while maintaining growth, as it was previously reported that plants mainly metabolize Na^+^ by ion exclusion from roots to rhizosphere, ion transportation in the vascular system, and its partition among tissues (Guo R. et al., 2015). To adapt to high salinity, plants prevent excessive accumulation of Na^+^ via two major approaches: exclusion from root uptake and vacuolar Na^+^ sequestration in the cytosol of root cells (Zhao et al., 2020). Under salt stress, therefore, the Na^+^ concentration in sodic soil can be used as a suitable indicator for predicting growth performance after early establishment of *B. planiculmis*.

Damage to plants due to high salinity includes a reduction in photosynthesis, leaf expansion, and ultimately biomass loss (Rahnama et al., 2010). Moreover, it has been indicated that salt stress may influence plant growth indirectly by lowering the photosynthesis rate (Brugnoli and Lauteri, 1991). In our study, the multiple linear regressions showed that the photosynthetic rate and leaf area were responsible for shoot biomass accumulation. The rapid stomatal closure induced by osmotic stress and the accumulation of high levels of salt ions in the cytosol resulted in photosynthetic efficiency limitations and net CO_2_ assimilation, and therefore a reduction in plant growth (Zhao et al., 2020). A substantial decrement in the photosynthesis of plants subjected to salt stress has been associated with a reduction in stomatal conductance and transpiration rates (Ribeiro et al., 2009), which likely explains the lower photosynthetic rates of *B. planiculmis* leaves under high level of salt-alkaline stress in this study. This is also supported by the fact that the greater sensitivity of photosynthesis in *B. maritimus* to salt is due to the greater sensitivity of its stomata to changing water potentials, resulting in decreased photosynthesis rates and stomatal closure (Maricle and Maricle, 2018). Furthermore, it is noted that less expanded leaves could possibly decrease water absorption by plants, thus allowing them to conserve relative moisture and prevent further enhancement in the salt level in the soil (Hanin et al., 2016). The stress-induced decrement in shoot growth of the *B. planiculmis* seedlings is largely attributed to the inhibition of leaf area development caused by salt-alkaline mixed stress. This is consistent with previous studies in which the substantial contribution of leaf expansion to biomass accumulation is more sensitive to stress (Parida and Das, 2005; Bernstein et al., 2010). The restriction in leaf area caused by salinity stress is thought to be a crucial approach to suppressing net photosynthetic rates and hence decreasing the available assimilates for leaf growth and biomass production (Rouphael et al., 2012). Therefore, photosynthetic rate and leaf area can be considered as the primary processes of *B. planiculmis* shoot growth affected by salt-alkaline stress. Variations in these traits are especially useful for elucidating the response and adaptation strategies of the aerial part of *B. planiculmis*.

Root architecture can represent attributes of the root system in terms of resource acquisition and exploration efficiency, especially under adverse conditions, as its direct relation with plant growth and productivity has been proven in a previous study (Lynch, 1995). The root growth of *B. planiculmis* seedlings acclimated substantially to the lower salinity and alkalinity conditions in this study. This may be explained by the fact that *B. planiculmis* appears to be more salt-tolerant than other freshwater species, which is consistent with its occurrence in alkaline water (Hroudová et al., 1999). As the salinity and alkalinity increased, the root length, root surface area, and root tip number of the *B. planiculmis* seedlings in this study decreased dramatically due to the ion toxicity resulting from the high salt concentration, which could restrict root extension (Neumann et al., 1999). The increased root length and number of root tips represent an effective strategy to increase root surface area, which improved the plant’s capacity for water and nutrient uptake. However, soil nutrient supply and ion balance might be disturbed when the salinity or pH reach high levels (Shi and Sheng, 2005). Otherwise, *B. planiculmis* plants increased root tip numbers when the root system was exposed to lower salt-alkaline stress, which may be an adaptive strategy to dehydration that improves its capacity for water and nutrient uptake. The lateral root formation was significantly altered under elevated salt to cope with stress (Waisel and Breckle, 1987). Thus, the root surface area and root tip number acted as the foremost determinants for root biomass accumulation of *B. planiculmis* under salt-alkaline stress.

## Conclusion

The seedling metrics of *B. planiculmis* under lower levels of salinity and alkalinity were promoted to some extent, suggesting that his plant can adapt well to salt-alkaline condition. PCA indicated that the stress effect of salinity on the early seedling growth of *B. planiculmis* was generally greater than that of alkalinity. Emergence and seedling growth of *B. planiculmis* had different responses to salt-alkaline stress. In particular, the seedling emergence was highly alkaline-dependent. Among the salt factors, Na^+^ played a prominent role in affecting seedling traits. The *B. planiculmis* seedlings adapted to saline-alkaline conditions by regulating their leaf and root traits. The variations in physiological and morphological characteristics could be used to explore the response and adaptation strategies of *B. planiculmis* plants to salt-alkaline stress. This study provided critical guidelines for exploring early establishment and distribution of *B. planiculmis* populations in wetlands and evaluating the restoration potential of degraded saline-sodic wetlands on the Songnen Plain.

## Data Availability Statement

The original contributions presented in the study are included in the article/supplementary material, further inquiries can be directed to the corresponding author/s.

## Author Contributions

YA and YG designed the experiment and analyzed the data. YA, YG, ST, and BL performed the experiments and wrote the manuscript. All the authors contributed to the article and approved the submitted version.

## Conflict of Interest

The authors declare that the research was conducted in the absence of any commercial or financial relationships that could be construed as a potential conflict of interest.

## References

[B1] An Y. Gao Y. Tong S. (2018). Emergence and growth performance of *Bolboschoenus planiculmis* varied in response to water level and soil planting depth: implications for wetland restoration using tuber transplantation. *Aquat. Bot.* 148 10–14. 10.1016/j.aquabot.2018.04.005

[B2] An Y. Gao Y. Zhang Y. Tong S. Liu X. (2019). Early establishment of *Suaeda salsa* population as affected by soil moisture and salinity: implications for pioneer species introduction in saline-sodic wetlands in Songnen Plain. China. *Ecol. Ind.* 107:105654. 10.1016/j.ecolind.2019.105654

[B3] Bernstein N. Kravchik M. Dudai N. (2010). Salinity-induced changes in essential oil, pigments and salts accumulation in sweet basil (*Ocimum basilicum*) in relation to alterations of morphological development. *Ann. Appl. Biol.* 156 167–177. 10.1111/j.1744-7348.2009.00376.x

[B4] Bose J. Rodrigo-Moreno A. Shabala S. (2014). ROS homeostasis in halophytes in the context of salinity stress tolerance. *J. Exp. Bot.* 65 1241–1257. 10.1093/jxb/ert430 24368505

[B5] Brugnoli E. Lauteri M. (1991). Effects of salinity on stomatal conductance, photosynthetic capacity, and carbon isotope discrimination of salt-tolerant (*Gossypium hirsutum* L.) and salt-sensitive (*Phaseolus vulgaris* L.) C(3) non-halophytes. *Plant Physiol.* 95 628–635. 10.1104/pp.95.2.628 16668029 PMC1077578

[B6] Chen Y. Li Y. Sun P. Chen G. Xin J. (2017). Interactive effects of salt and alkali stresses on growth, physiological responses and nutrient (N, P) removal performance of *Ruppia maritima*. *Ecol. Eng.* 104 177–183. 10.1016/j.ecoleng.2017.04.029

[B7] Chi C. M. Zhao C. W. Sun X. J. Wang Z. C. (2012). Reclamation of saline-sodic soil properties and improvement of rice (*Oryza sativa* L.) growth and yield using desulfurized gypsum in the west of Songnen Plain, northeast China. *Geoderma* 18 24–30. 10.1016/j.geoderma.2012.04.005

[B8] de Lacerda C. F. Cambraia J. Oliva M. A. Ruiz H. A. Prisco J. T. (2003). Solute accumulation and distribution during shoot and leaf development in two sorghum genotypes under salt stress. *Environ. Exp. Bot.* 49 107–120. 10.1016/s0098-8472(02)00064-3

[B9] Deinlein U. Stephan A. B. Horie T. Luo W. Xu G. Schroeder J. I. (2014). Plant salt-tolerance mechanisms. *Trends Plant Sci.* 19 371–379. 24630845 10.1016/j.tplants.2014.02.001 PMC4041829

[B10] Ebrahimi E. Eslami S. V. (2012). Effect of environmental factors onseed germination and seedling emergence of invasive *Ceratocarpus arenarius*. *Weed Res*. 52 50–59. 10.1111/j.1365-3180.2011.00896.x

[B11] Elmore A. J. Manning S. J. Mustard J. F. Craine J. M. (2006). Decline in alkali meadow vegetation cover in California: the effects of groundwater extraction and drought. *J. Appl. Ecol.* 43 770–779. 10.1111/j.1365-2664.2006.01197.x

[B12] Engels J. G. Rink F. Jensen K. (2011). Stress tolerance and biotic interactions determine plant zonation patterns in estuarine marshes during seedling emergence and early establishment. *J. Ecol.* 99 277–287. 10.1111/j.1365-2745.2010.01745.x

[B13] Flowers T. J. Colmer T. D. (2008). Salinity tolerance in halophytes. *New Phytol.* 179 945–963. 10.1111/j.1469-8137.2008.02531.x 18565144

[B14] Galvan-Ampudia C. S. Julkowska M. M. Darwish E. Gandullo J. Korver R. A. Brunoud G. (2013). Halotropism is a response of plant roots to avoid a saline environment. *Curr. Biol.* 23 2044–2050. 10.1016/j.cub.2013.08.042 24094855

[B15] Ge Y. Li J. D. (1990). A preliminary study on the effects of halophyteson salt accumulation and desalination in the soil of Songnen Plain, northeast China. *Acta Pratacult. Sin.* 1 70–76.

[B16] Ghoulam C. Foursy A. Fares K. (2002). Effects of salt stress on growth, inorganic ions and proline accumulation in relation to osmotic adjustment in five sugar beet cultivars. *Environ. Exp. Bot.* 47 39–50. 10.1016/s0098-8472(01)00109-5

[B17] Grewal H. S. (2010). Response of wheat to subsoil salinity and temporary water stress at different stages of the reproductive phase. *Plant Soil* 330 103–113. 10.1007/s11104-009-0179-7

[B18] Guo C. Y. Wang X. Z. Chen L. Ma L. N. Wang R. Z. (2015). Physiological and biochemical responses to saline-alkaline stress in two halophytic grass species with different photosynthetic pathways. *Photosynthetica* 53 128–135. 10.1007/s11099-015-0094-5

[B19] Guo R. Yang Z. Li F. Yan C. Zhong X. Liu Q. (2015). Comparative metabolic responses and adaptive strategies of wheat (*Triticum aestivum*) to salt and alkali stress. *BMC Plant Biol*. 15:170. 10.1186/s12870-015-0546-x 26149720 PMC4492011

[B20] Hanin M. Ebel C. Ngom M. Laplaze L. Masmoudi K. (2016). New insights on plant salt tolerance mechanisms and their potential use for breeding. *Front Plant Sci.* 7:1787. 10.3389/fpls.2016.01787 27965692 PMC5126725

[B21] Hroudová Z. Zákravskı P. Ducháèek M. Marhold K. (2007). Taxonomy, distribution and ecology of *Bolboschoenus* in Europe. *Ann. Bot. Fenn.* 44 81–102.

[B22] Hroudová Z. Zákravskı P. Flegrová M. (2014). The tolerance to salinity and nutrient supply in four European *Bolboschoenus* species (*B. maritimus*, *B. laticarpus*, *B. planiculmis* and *B. yagara*) affects their vulnerability or expansiveness. *Aquat. Bot.* 112 66–75. 10.1016/j.aquabot.2013.07.012

[B23] Hroudová Z. Zákravskı P. Frantík T. (1999). Ecological differentiation of Central European *Bolboschoenus* taxa and their relationship to plant communities. *Folia Geobot.* 34 77–96. 10.1007/bf02803077

[B24] Jenačković D. D. Zlatković I. D. Lakušić D. V. Ranðelović V. N. (2016). Macrophytes as bioindicators of the physicochemical characteristics of wetlands in lowland and mountain regions of the central Balkan Peninsula. *Aquat. Bot.* 134 1–9. 10.1016/j.aquabot.2016.06.003

[B25] Khajeh-Hosseini M. Powell A. A. Bingham I. J. (2003). The interaction between salinity stress and seed vigour during germination of soybean seeds. *Seed Sci. Technol.* 31 715–725. 10.15258/sst.2003.31.3.20

[B26] Li R. Shi F. Fukuda K. (2010). Interactive effects of various salt and alkali stresses on growth, organic solutes, and cation accumulation in a halophyte *Spartina alterniflora* (*Poaceae*). *Environ. Exp. Bot.* 68 66–74. 10.1016/j.envexpbot.2009.10.004

[B27] Liu B. Kang C. Wang X. Bao G. (2015). Tolerance mechanisms of *Leymus chinensis* to salt-alkaline stress. *Acta Agr. Scand. B Soil Plant Sci.* 65 723–734.

[B28] Liu Q. Cui B. Yang Z. (2009). Dynamics of the soil water and solute in the sodic saline soil in the Songnen Plain. *China. Environ. Earth Sci.* 59 837–845. 10.1007/s12665-009-0079-4

[B29] Liu Y. Ding Z. Bachofen C. Lou Y. Jiang M. Tang X. (2018). The effect of saline-alkaline and water stresses on water use efficiency and standing biomass of *Phragmites australis* and *Bolboschoenus planiculmis*. *Sci. Total Environ.* 644 207–216. 10.1016/j.scitotenv.2018.05.321 29981969

[B30] Ljevnaić-Mašić B. Džigurski D. Nikolić L. Brdar-Jokanović M. Čabilovski R. Ćirić V. (2020). Assessment of the habitat conditions of a rare and endangered inland saline wetland community with *Bolboschoenus maritimus* (L.) Palla dominance in Southeastern Europe: the effects of physical–chemical water and soil properties. *Wetl. Ecol. Manag.* 28 421–438. 10.1007/s11273-020-09721-4

[B31] Lowe B. J. Watts R. J. Roberts J. Robertson A. (2010). The effect of experimental inundation and sediment deposition on the survival and growth of two herbaceous riverbank plant species. *Plant Ecol.* 209 57–69. 10.1007/s11258-010-9721-1

[B32] Lv B. S. Li X. W. Ma H. Y. Sun Y. Wei L. X. Jiang C. J. (2013). Differences in growth and physiology of rice in response to different saline-alkaline stress factors. *Agron. J.* 105 1119–1128. 10.2134/agronj2013.0017

[B33] Lynch J. (1995). Root architecture and plant productivity. *Plant Physiol.* 109 7–13. 10.1104/pp.109.1.7 12228579 PMC157559

[B34] Ma H. Yang H. Lü X. Pan Y. Wu H. Liang Z. (2015). Does high pH give a reliable assessment of the effect of alkaline soil on seed germination? A case study with *Leymus chinensis* (*Poaceae*). *Plant Soil* 394 35–43. 10.1007/s11104-015-2487-4

[B35] Maricle B. R. Maricle K. L. (2018). Photosynthesis, stomatal responses, and water potential in three species in an inland salt marsh in Kansas. USA. *Flora* 244 1–7. 10.1016/j.flora.2018.05.001

[B36] Mony C. Puijalon S. Bornette G. (2011). Resprouting response of aquatic clonal plants to cutting may explain their resistance to spate flooding. *Folia Geobot.* 46 155–164. 10.1007/s12224-010-9095-0

[B37] Morris K. Ganf G. G. (2001). The response of an emergent sedge *Bolboschoenus medianus* to salinity and nutrients. *Aquat. Bot.* 70 311–328. 10.1016/s0304-3770(01)00152-8

[B38] Nakamura I. Hossain M. A. (2009). Factors affecting the seed germination and seedling emergence of red flower ragleaf (*Crassocephalum crepidioides*). *Weed Biol. Manag.* 9 315–322. 10.1111/j.1445-6664.2009.00356.x

[B39] Neumann G. Massonneau A. Martinoia E. Romheld V. (1999). Physiological adaptations to phosphorus deficiency during proteoid root development in white lupin. *Planta* 208 373–382. 10.1007/s004250050572

[B40] Ning Y. Zhang Z. X. Cui L. J. Zou C. L. (2014). Adaptive significance of and factors affecting plasticity of biomass allocation and rhizome morphology: a case study of the clonal plant *Scirpus planiculmis* (*Cyperaceae*). *Pol. J. Ecol.* 62 77–88. 10.3161/104.062.0108

[B41] Parida A. K. Das A. B. (2005). Salt tolerance and salinity effects on plants: a review. *Ecotox. Environ. Saf.* 60 324–349. 10.1016/j.ecoenv.2004.06.010 15590011

[B42] Píšová S. Hroudova Z. Chumova Z. Fer T. (2017). Ecological hybrid speciation in central-European species of *Bolboschoenus*: genetic and morphological evaluation. *Preslia* 89 17–39. 10.23855/preslia.2017.017

[B43] Rahnama A. James R. A. Poustini K. Munns R. (2010). Stomatal conductance as a screen for osmotic stress tolerance in durum wheat growing in saline soil. *Funct. Plant Biol*. 37 255–263. 10.1071/fp09148

[B44] Ribeiro R. V. Machado E. C. Santos M. G. Oliveira R. F. (2009). Photosynthesis and water relations of well-watered orange plants as affected by winter and summer conditions. *Photosynthetica* 47 215–222. 10.1007/s11099-009-0035-2

[B45] Rouphael Y. Cardarelli M. Rea E. Colla G. (2012). Improving melon and cucumber photosynthetic activity, mineral composition, and growth performance under salinity stress by grafting onto *Cucurbita* hybrid rootstocks. *Photosynthetica* 50 180–188. 10.1007/s11099-012-0002-1

[B46] Shi D. Sheng Y. (2005). Effect of various salt–alkaline mixed stress conditions on sunflower seedlings and analysis of their stress factors. *Environ. Exp. Bot.* 54 8–21. 10.1016/j.envexpbot.2004.05.003

[B47] Shi D. C. Wang D. L. (2005). Effects of various salt–alkaline mixed stresses on *Aneurolepidium chinense* (Trin.) Kitag. *Plant Soil* 271 15–26. 10.1007/s11104-004-1307-z

[B48] Silvestri S. Defina A. Marani M. (2005). Tidal regime, salinity and salt marsh plant zonation. *Estuar. Coast. Shelf Sci*. 62 119–130. 10.1016/j.ecss.2004.08.010

[B49] Sosa L. LLanes A. Reinoso H. Reginato M. Luna V. (2005). Osmotic and specific ion effects on the germination of *Prosopis strombulifera*. *Ann. Bot.* 96 261–267. 10.1093/aob/mci173 15928009 PMC4246873

[B50] Sosnová M. van Diggele R. Klimešová J. (2010). Distribution of clonal growth forms in wetlands. *Aquat. Bot.* 92 33–39. 10.1016/j.aquabot.2009.09.005

[B51] Stokes C. A. MacDonald G. E. Adams C. R. Langeland K. A. Miller D. L. (2011). Seed biology and ecology of natalgrass (*Melinis repens*). *Weed Sci.* 59 527–532. 10.1614/ws-d-11-00028.1

[B52] Tang C. Turner N. C. (1999). The influence of alkalinity and water stress on the stomatal conductance, photosynthetic rate and growth of *Lupinus angustifolius* L. and *Lupinus pilosus* Murr. *Aust. J. Exp. Agric.* 39 457–464. 10.1071/ea98132

[B53] Waisel Y. Breckle S. W. (1987). Differences in responses of various radish roots to salinity. *Plant Soil* 104 191–194. 10.1007/bf02372532

[B54] Wang L. Seki K. Miyazaki T. Ishihama Y. (2009). The causes of soil alkalinization in the Songnen Plain of Northeast China. *Paddy Water. Environ.* 7 259–270. 10.1007/s10333-009-0166-x

[B55] Wang S. Zheng W. Ren J. Zhang C. (2002). Selectivity of various types of salt-resistant plants for K^+^ over Na^+^. *J. Arid Environ.* 52 457–472. 10.1006/jare.2002.1015

[B56] Wang X. Cheng R. Zhu H. Cheng X. Shutes B. Yan B. (2020). Seed germination and early seedling growth of six wetland plant species in saline-alkaline environment. *Int. J. Phytoremediat.* 22 1185–1194. 10.1080/15226514.2020.1748565 32281893

[B57] Yang C. W. Jianaer A. Li C. Y. Shi D. C. Wang D. L. (2008). Comparison of the effects of salt-stress and alkali-stress on photosynthesis and energy storage of an alkali-resistant halophyte *Chloris virgata*. *Photosynthetica* 46 273–278.

[B58] Yang C. W. Xu H. H. Wang L. L. Liu J. Shi D. C. Wang D. L. (2009). Comparative effects of salt-stress and alkali-stress on the growth, photosynthesis, solute accumulation, and ion balance of barley plants. *Photosynthetica* 47 79–86. 10.1007/s11099-009-0013-8

[B59] Yang J. Zhang S. Li Y. Bu K. Zhang Y. Chang L. (2010). Dynamics of saline-alkali land and its ecological regionalization in western Songnen Plain. *China. Chin. Geogr. Sci.* 20 159–166. 10.1007/s11769-010-0159-0

[B60] Yang J. Y. Zheng W. Tian Y. Wu Y. Zhou D. W. (2011). Effects of various mixed salt-alkaline stresses on growth, photosynthesis, and photosynthetic pigment concentrations of *Medicago ruthenica* seedlings. *Photosynthetica* 49 275–284. 10.1007/s11099-011-0037-8

[B61] Zhang H. Irving L. J. McGill C. Matthew C. Zhou D. Kemp P. (2010). The effects of salinity and osmotic stress on barley germination rate: sodium as an osmotic regulator. *Ann. Bot.* 106 1027–1035. 10.1093/aob/mcq204 20929898 PMC2990672

[B62] Zhang H. Tian Y. Guan B. Zhou D. Sun Z. Baskin C. C. (2018). The best salt solution parameter to describe seed/seedling responses to saline and sodic salts. *Plant Soil* 426 313–325. 10.1007/s11104-018-3623-8

[B63] Zhang L. Zhang G. Li H. Sun G. (2014). Eco-physiological responses of *Scirpus planiculmis* to different water-salt conditions in Momoge Wetland. *Pol. J. Environ. Stud.* 23 1813–1820.

[B64] Zhang W. Yang G. Sun J. Chen J. Zhang Y. (2015). Clonal integration enhances the performance of a clonal plant species under soil alkalinity stress. *PLoS One* 10:e0119942. 10.1371/journal.pone.0119942 25790352 PMC4366383

[B65] Zhao C. Zhang H. Song C. Zhu J. K. Shabala S. (2020). Mechanisms of plant responses and adaptation to soil salinity. *Innovation* 1:100017. 10.1016/j.xinn.2020.100017 PMC8454569 34557705

[B66] Zhao Y. Lu Z. He L. (2014). Effects of saline-alkaline stress on seed germination and seedling growth of *Sorghum bicolor* (L.) Moench. *Appl. Biochem. Biotechnol.* 173 1680–1691. 10.1007/s12010-014-0956-5 24840039

